# The History of Jaundice in Bristol

**Published:** 1966-10

**Authors:** Bimla Arora

**Affiliations:** Department of Medicine, United Bristol Hospitals


### THE HISTORY OF JAUNDICE IN BRISTOL

BY

BIMLA ARORA

*(Department of Medicine, United Bristol Hospitals)*

*"Much can be learned from these ancient writings, but much must be left to conjecture."*—Dr. W. N. Pickles.

Writers of the history of Bristol have paid scant attention to the occurrence of any disease or epidemic in the city, although recently an excellent account of plague in the XVIIth century has been published (Hemphill, 1966).

An interest in the recent epidemic of infective hepatitis in Bristol led the present writer to look for evidence for the existence of cases of jaundice in the past, and especially to determine whether there had been any previous epidemics in which jaundice was the main feature. The City Archives have no record of any such epidemic. But the Bristol Infirmary (as it then was) possesses a collection of old clinical records, and all the volumes that were available have been examined.

The information that can be obtained from the old Infirmary records—both the out-patient registers and the accounts of the patients that were admitted to the beds of the charitable institution—at first appears meagre; but for determining the occurrence of jaundice there is sufficient evidence. The earliest recorded case of jaundice was in 1739, when the records began; in that year three persons with jaundice were seen as out-patients, and from then onwards, whenever written evidence is available, there seem to have been a few cases each year.

The old volumes provide each patient's name, his age, his parish of residence, the name of the doctor or other functionary who recommended him, the date when he was seen as an out-patient and admitted to or discharged from a hospital bed, and the nature of his distemper. On leaving the Infirmary a patient was either " relieved " or " cured " of the ailment (though some patients evidently " absconded "), and during the XVIIIth century the remainder left as " incurable ". It is only in the next century that " death " in some cases has been noted as the outcome. Some of the writing was difficult to read, on some pages there were smudges, and the registers had obviously survived the hazards of flooding, burning, and wars; yet it has been possible to survey the period from 1741 to 1855 fairly completely.

Those cases have been considered in which the distemper was diagnosed as " icterus ", " icterus morbus ", " hepatitis ", or simply " jaundice ". Those labelled " scirrhous " have also been included; they might have been regarded as cases of cirrhosis of the liver, but the patients were mostly young and of either sex, and in any case they were few in number. It was not possible to determine the cause of the jaundice in any of these cases, for instance, it is possible that Weil's disease, typhus, or syphilis may have contributed to the total. " Inveterate itch " accompanied the jaundice in some patients, and these cases have been included because, although the jaundice may have been obstructive or neoplastic, most of the patients were young adults, and the great majority recovered; it is therefore likely that in most cases the illness was infective hepatitis.

Table I shows the information that has been assembled. It refers to patients who were admitted to the Infirmary and shows, for each 5-year period, the average number of cases of jaundice per year. From 1753 to 1762 there were more cases than in the preceding or following years, and there was another increase in the period 1816 to 1820. A particularly high admission rate marked the years 1853 and 1854 (which are shown separately in the Table). Total yearly admissions for all causes are not shown, for although they increased slightly over the period concerned, they did not fluctuate as the admissions for jaundice did. From these figures it may be inferred that jaundice (probably infective hepatitis) was present in Bristol during the XVIIIth and XIXth centuries, and that at some periods it was fairly common in the city.

The registers provide ample evidence that other communicable diseases, such as typhus and cholera, occurred during the first half of the XIXth century, as would be expected in an overcrowded city with poor sanitation and an impure water supply (Latimer, 1887).

TABLE I

Cases of jaundice admitted to hospital in Bristol, 1741 to 1855.

| 5-year period | Average admissions per year | 5-year period | Average admissions per year |
|---|---|---|---|
| 1741-45 | 1 | 1826-30 | 11.4 |
| 1753-57 | 11.2 | 1831-35 | 8 |
| 1758-62 | 18.6 | 1836-40 | 8 |
| | | 1841-45 | 8.8 |
| 1801-05 | 3.6 | | |
| 1806-10 | 4.2 | 1853 | 22 |
| 1811-15 | 6 | 1854 | 37 |
| 1816-20 | 14.4 | 1855 | 14 |
| 1821-25 | 10 | | |

Of course, no direct epidemiological comparison can be made between these early records and those of the present day; for the present century has seen enormous changes in the differential diagnosis of jaundice, a great increase in the number of hospitals in Bristol, a change in the usage of hospital beds, and new methods of case-finding and case-recording. But one comparison of possible significance may be made: in 1854 there were 37 patients with jaundice admitted to the Infirmary, of whom 4 died; more than a century later, in the peak years (1960 and 1961) of the recent epidemic of infective hepatitis only 22 patients were admitted for this disease, and of these 2 died. In this same 2-year period 2,107 cases of infective hepatitis were notified in Bristol (Bothwell *et al.*, 1963). Hospital admissions represent (in this century) only a tiny fraction of the incidence of this disease in the community.

This search of the old records was undertaken to provide a historical background for the study of the recent epidemic of infective hepatitis. It is now apparent that similar epidemics may have occurred in the city in the past.

*(This paper is drawn from the material for a dissertation, the title of which has been accepted by the University of Bristol for the degree of M.D.)*

REFERENCES

Bothwell, P. W., Martin, D., Macara, A. W., Skone, J. F. and Wofinden, R. C. (1963). *Brit. Med. Jl.*, **ii**, 1613.

Hemphill, R. E. (1966). *Bristol Med. Chi. Jl.*, **81**, 1.

Latimer, J. (1887). "Annals of Bristol in the 19th Century".

